# Zinc ion implantation-deposition technique improves the osteoblast biocompatibility of titanium surfaces

**DOI:** 10.3892/mmr.2015.3311

**Published:** 2015-02-06

**Authors:** YONGQIANG LIANG, JUAN XU, JING CHEN, MENGCHUN QI, XUEHONG XIE, MIN HU

**Affiliations:** 1 College of Stomatology, Hebei United University, Tangshan, Hebei 063000, P.R. China; 2 Department of Stomatology, Chinese People’s Liberation Army General Hospital, Beijing 100853, P.R. China; 3 College of Life Sciences, Hebei United University, Tangshan, Hebei 063000, P.R. China

**Keywords:** zinc implantation-deposition, titanium surface modification, MG-63 cells, biocompatibility

## Abstract

The plasma immersion ion implantation and deposition (PIIID) technique was used to implant zinc (Zn) ions into smooth surfaces of pure titanium (Ti) disks for investigation of tooth implant surface modification. The aim of the present study was to evaluate the surface structure and chemical composition of a modified Ti surface following Zn ion implantation and deposition and to examine the effect of such modification on osteoblast biocompatibility. Using the PIIID technique, Zn ions were deposited onto the smooth surface of pure Ti disks. The physical structure and chemical composition of the modified surface layers were characterized by scanning electron microscopy (SEM) and X-ray photoelectron spectroscopy (XPS), respectively. *In vitro* culture assays using the MG-63 bone cell line were performed to determine the effects of Zn-modified Ti surfaces following PIIID on cellular function. Acridine orange staining was used to detect cell attachment to the surfaces and cell cycle analysis was performed using flow cytometry. SEM revealed a rough ‘honeycomb’ structure on the Zn-modified Ti surfaces following PIIID processing and XPS data indicated that Zn and oxygen concentrations in the modified Ti surfaces increased with PIIID processing time. SEM also revealed significantly greater MG-63 cell growth on Zn-modified Ti surfaces than on pure Ti surfaces (P<0.05). Flow cytometric analysis revealed increasing percentages of MG-63 cells in S phase with increasing Zn implantation and deposition, suggesting that MG-63 apoptosis was inhibited and MG-63 proliferation was promoted on Zn-PIIID-Ti surfaces. The present results suggest that modification with Zn-PIIID may be used to improve the osteoblast biocompatibility of Ti implant surfaces.

## Introduction

In previous years, implanted prosthodontic dentures have been applied as an effective way to repair either defective or absent dentition. The surface characteristics and morphology of implanted dentures affect the cell survival, adhesion, proliferation and differentiation of dental tissues on denture surfaces (1). Biologically inert titanium (Ti) is widely used in dental implants due to the direct contact between the implant material and the bone (1,2). However, as a bioinert material, Ti cannot be biologically integrated with bone tissue (3). One approach to improve integration has been to roughen endosseous areas of dental implants in order to increase the total surface area available for osseous apposition. Surface roughness has been demonstrated to alter osteoblast attachment, proliferation, differentiation and extracellular matrix (ECM) production (4).

Another limitation of Ti implants is the short delay between implantation and when they are able to impart bactericidal capabilities, which may lead to the formation of dental plaques surrounding implanted dentures (5). Several strategies have been developed to overcome this surface-associated limitation of Ti implants (6–10), including surface modification by plasma immersion ion implantation and deposition (PIIID) (11–13). PIIID generates surface layers that can integrate with specific substrates and this technique has been used to incorporate zinc (Zn) into the surfaces of Ti implants (14). Zn is a necessary element for cellular activities and is specifically important in the development and maintenance of bone structures (15).

In the current study, a PIIID technique was used to implant Zn ions onto the smooth surfaces of pure Ti disks. The physical structure and chemical composition of the modified Zn-PIIID-Ti surfaces were evaluated by scanning electron microscopy (SEM) and X-ray photoelectron spectroscopy (XPS), respectively and then the biocompatibility of the Zn-PIIID-Ti surfaces with regard to osteoblast function *in vitro* was examined.

## Materials and methods

### Preparation and surface characterization of Ti disks

Pure Ti (grade 4) disks, 10 mm in diameter and 1 mm thick were polished on one side to a mirror-like finish and then sequentially subjected to ultrasonication in acetone, absolute alcohol and deionized water. The clean disks were then air-dried and stored in a desiccator. PIIID to implant and deposit Zn ions was performed at the State Key Laboratory of Advanced Welding Production Technology of the Harbin Institute of Technology (Harbin, China) using the following parameters: Pulse voltage (V) of 20 kv; pulse width (τ/) of 300 *μ*s; Zn-ion pulsed cathodic arc width of 300 *μ*s and working pressure (P) of 1×10^−1^ Pa. The source of the Zn ions was a pulsed cathodic arc. Ti disks were prepared with four different implantation times (T) of 20 min (Zn-Ti-20 min group), 40 min (Zn-Ti-40 min group), 60 min (Zn-Ti-60 min group) and 80 min (Zn-Ti-80 min group). Commercially pure Ti (cp-Ti) disks without Zn implantation were used in the control group.

The physical structure of modified Ti disks was observed by SEM (Hitachi S-520; Hitachi, Ltd., Tokyo, Japan) and their chemical composition was characterized by XPS using 300 W Al Kα radiation and an ESCALab 220i-XL electron spectrometer (VG Scientific, Ltd., East Grinstead, UK). Binding energies were referenced to the C1s line at 284.8 eV from trace carbon and the base pressure was 3×10^−9^ mbar.

### Cell culture

MG-63 human osteosarcoma cells (purchased from the Cancer Institute and Hospital, Chinese Academy of Medical Sciences, Beijing, China) were grown in minimum essential medium with 10% fetal calf serum (HyClone Laboratories, Inc., Logan, UT, USA), 100 IU/ml penicillin, 100 IU/ml streptomycin (North China Pharmaceutical Group Corporation, Shijiazhuang, China) and 2 mM L-glutamine in a humidified atmosphere of 5% CO_2_ at 37˚C. Cells were seeded onto surfaces of Zn-modified Ti disks in the Zn-Ti-20, −40, −60 and −80 min groups as well as control disks in 24-well plates at a seeding concentration of 2×10^4^ cells/ml.

### SEM observation of cell attachment

The morphology of attached cells was observed by SEM (Hitachi S-520; Hitachi, Ltd.). Following culture for 48 h, the Zn-modified and control Ti disks with attached cells were washed with phosphate-buffered saline (PBS) and the cells were fixed overnight in 2.5% glutaraldehyde. Following fixation, the samples were sequentially dehydrated (10 min each) in increasing concentrations of ethanol (20, 50, 70, 90 and 100%). Following dehydration, they were immersed in isoamyl acetate (a critical point drying fluid) for 1.5 min and finally sputter-coated with a thin layer of gold/palladium for viewing by SEM.

### Quantification of cell attachment and proliferation

Cell attachment to the Zn-modified and control Ti disks was quantified using acridine orange (AO) staining. Following a specific incubation time (6, 24 or 48 h), samples were fixed in 95% ethanol and stained with 4×10^4^ mg/ml AO for 1 min. Following rinsing with PBS, the samples were examined under a fluorescence microscope (FV1000; Olympus, Tokyo, Japan) and the attached cells were counted in randomly selected 2 mm^2^ areas.

### Flow cytometry for cell cycle analysis

After 48 h, MG-63 cells were collected and prepared for flow cytometry (FACSCalibur, Becton Dickinson Immunocytometry Systems, San Jose, CA, USA). A total of 10,000 cells were counted per sample and the fractions of cells in the G1, S and G2 phases of the cell cycle were determined. This experiment was performed in triplicate.

### Statistical analysis

Data are expressed as the mean ± standard deviation. Statistical analyses were performed using SPSS 12.0 software (SSPS, Inc., Chicago, IL, USA). One-way analysis of variance was conducted to assess differences among all quantitative indices. P<0.05 was considered to indicate a statistically significant difference.

## Results

### Structure and chemical composition of pure and Zn-modified Ti surfaces

**Figure 1:**
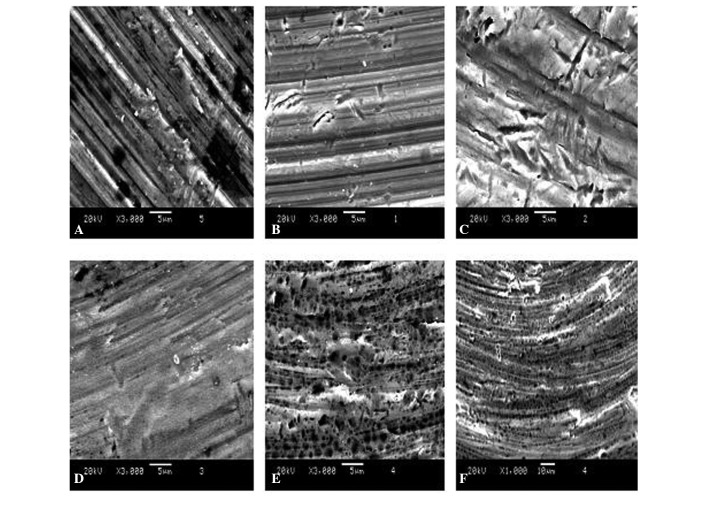
Scanning electron microscope images of unmodified and Zn-modified Ti surfaces. (A) Commercially pure-Ti, (B) Zn-Ti-20 min, (C) Zn-Ti-40 min, (D) Zn-Ti-60 min, (E) Zn-Ti-80 min and (F) Zn-Ti-80 min surfaces. (A-E) Magnification ×3,000, scale bar=5 *μ*m; (F) magnification ×1,000, scale bar=10 *μ*m. TI, titanium; Zn, zinc.

SEM images of the unmodified cp-Ti surfaces revealed tiny fissures and fuzzy grain edges with smooth shapes (Fig. 1). By contrast, multiple homogeneously and randomly distributed granular masses were apparent on the Zn-PIIID-Ti surfaces with increasing Zn exposure time. The Zn appeared to have penetrated into the base material and deposited onto the surface. For longer implantation and deposition times, the interstices among the distributed granular masses increased. The size and shape of the masses were also increasingly more homogeneous and the edges of the grains became much sharper, but no significant differences were observed in the number of minor fissures among the samples prepared with different exposure times (P>0.05). SEM images revealed that the Ti surfaces exhibited a rough ‘honeycomb’ structure in which the diameter was 60–100 nm with PIIID processing. The ‘honeycomb’ structure deepened with increasing processing time and surfaces in the Zn-Ti-80 min group demonstrated a 200 nm deep structure with grade 9 smoothness and 0.4 *μ*m roughness (Fig. 1F).

**Figure 2:**
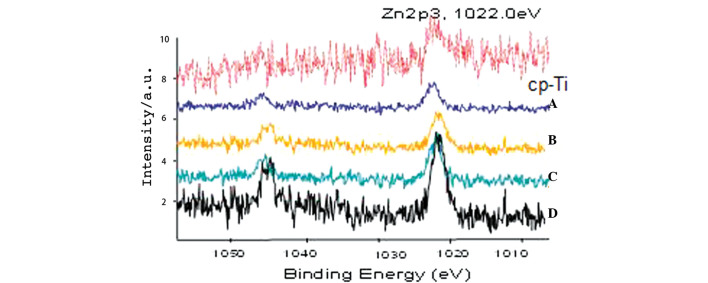
X-ray photoelectron spectra of unmodified (top) and Zn-modified Ti surfaces. (A) Zn-Ti-20 min, (B) Zn-Ti-40 min, (C) Zn-Ti-60 min and (D) Zn-Ti-80 min. TI, titanium; Zn, zinc.

The XPS results revealed alterations in the surface layer chemical composition upon Zn-PIIID (Fig. 2). The O1s peak relative intensity significantly decreased following PIIID. An additional peak corresponding to Zn-containing materials was also observed, although its intensity was extremely weak. XPS analysis also was used to obtain relative atomic concentrations of carbon, Ti and Zn on the surface of each group (Table I). In the Zn-Ti-20, −40, −60 and −80 min groups, the percentages of Zn were 1.04±0.08, 1.32±0.06, 1.35±0.04 and 2.14±0.06, respectively.

### MG-63 morphological alterations on pure and Zn-modified Ti surfaces

**Figure 3:**
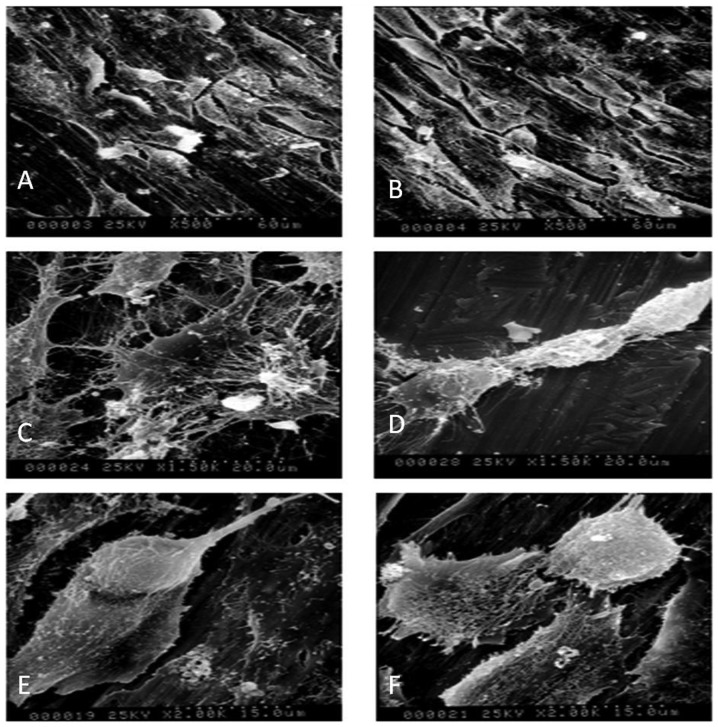
Scanning electron microscope images demonstrating the morphology of MG-63 cells on unmodified and Zn-modified Ti surfaces. (A and C) MG-63 cells on cp-Ti; (B and D-F) MG-63 cells on Zn-Ti-20 min surfaces. (C) MG-63 cells were primarily rounded on unmodified Ti surfaces, whereas on the (D) Zn-modified Ti surfaces, cells assumed fusiform shapes with well-spread pseudopodia and extensive networks of cytoplasmic processes. (D) Secreted extracellular matrix was visible covering the substrate granules and (E) cells undergoing division were observed. (A and B) Magnification, ×500. (C and D) Magnification, ×1,500. (E and F) Magnification, ×2,000. Cp, commercially pure; TI, titanium; Zn, zinc.

As shown in Fig. 3A, MG-63 cells typically have irregular polygon shapes, where the ratio of the macro axis to the minor axis is usually greater than those of other cells growing in the culture flask. SEM analysis revealed that the *in vitro* morphology of MG-63 cells was altered on Zn-PIIID-Ti surfaces (Fig. 3B). Compared with the predominately round cells observed on the cp-Ti disks (Fig. 3C), cells grown on the Zn-PIIID-Ti disks exhibited a relatively improved spreading and fattening, with an increased cell to substrate contact ratio (Fig. 3D). Increased Zn implantation resulted in a greater density of attached cells. The cells secreted considerable quantities of ECM protein that covered the granular surface (Fig. 3E) and appeared to be actively proliferating and differentiating (Fig. 3F). Cells on the Zn-PIIID-Ti surfaces also had extensive networks of cytoplasmic processes that extended to the underlying substrate and connected to neighboring cells, whereas cells on the cp-Ti surfaces had far fewer and shorter fibrillar extensions.

### Cell proliferation on pure and Zn-modified Ti surfaces\

**Figure 4:**
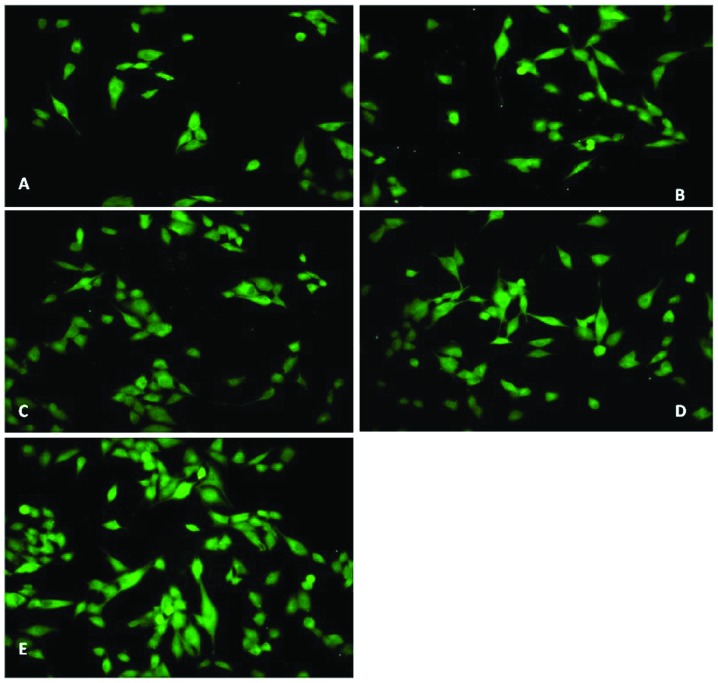
Fluorescence microscopy images of MG-63 cell proliferation on unmodified and Zn-modified Ti surfaces. Acridine orange-stained MG-63 cells on (A) cp-Ti, (B) Zn-Ti-20 min, (C) Zn-Ti-40 min, (D) Zn-Ti-60 min and (E) Zn-Ti-80 min surfaces after 24 h in culture. Cp, commercially pure; Ti, titanium; Zn, zinc

**Figure 5:**
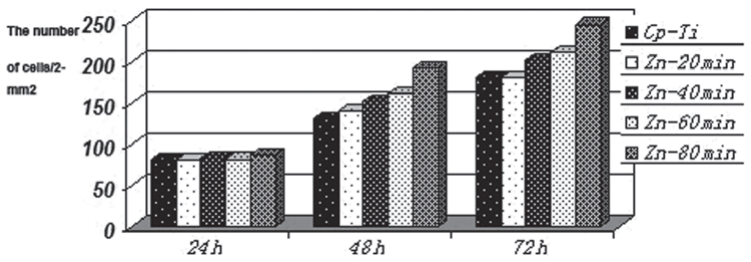
Number of MG-63 cells (per 2 mm^2^ area) observed on unmodified cp-Ti and Zn-modified Ti surfaces with increasing culture time (n=12). Cp, commercially pure; TI, titanium; Zn, zinc.

The initial attachment of cells is crucial to subsequent cell spreading, proliferation and differentiation on substrates. As shown in Fig. 4 by fluorescence microscopy, the number of attached and proliferating MG-63 cells increased with culture time for all samples. At 6 h after cell seeding, there were no significant differences identified in cell proliferation among the five samples (P>0.05). However, after 24 h, a higher density of cells was present on the Zn-PIIID-Ti surfaces compared with that on the cp-Ti surface (P<0.05). This trend became more pronounced for higher levels of Zn ion implantation and deposition, whereas no significant difference in the quantified numbers of cells was noted between the Zn-Ti-60 min and Zn-Ti-80 min groups (P>0.05; Fig. 5).

### MG-63 cell cycle distribution on pure and Zn-modified Ti surfaces

**Figure 6:**
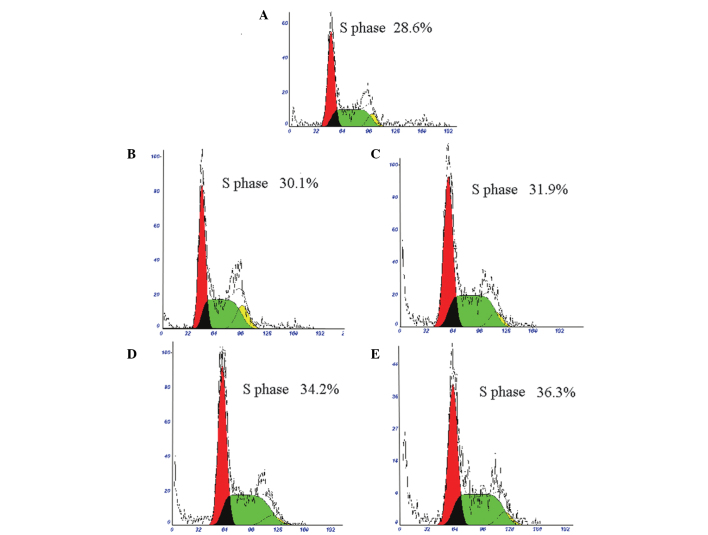
Cell cycle analysis of MG-63 cells on (A) commercially pure-Ti, (B) Zn-Ti-20 min, (C) Zn-Ti-40 min, (D) Zn-Ti-60 min and (E) Zn-Ti-80 min surfaces after 48 h in culture. Data represent numbers of cells per 2 mm^2^ area. Zn, zinc; Ti, titanium.

After 48 h in culture, flow cytometric analysis revealed that the percentage of cells in S phase was 28.62% in the cp-Ti group (Fig. 6A), 30.1% in the Zn-Ti-20 min group (Fig. 6B), 31.9% in the Zn-Ti-40 min group (Fig. 6C), 34.2% in the Zn-Ti-60 min group (Fig. 6D) and 36.3% in the Zn-Ti-80 min group (Fig. 6E). These percentages for each of the five groups were significantly different from each other (P<0.01) within this gradual increase in the S phase from the Zn-Ti-20 min group to the Zn-Ti-80 min group. These results demonstrated that the modified Zn-PIIID-Ti surfaces may inhibit MG-63 cell apoptosis as well as promote proliferation.

## Discussion

Previous studies have reported that increased surface roughness of Ti implants improves t he rate of osseointegration and biomechanical fixation (16–18). Surface roughness in the nanometer range is important in the absorption of proteins and adhesion of osteoblastic cells and thus affects the rate of osseointegration (19). However, surface roughness at this scale is difficult to achieve using chemical treatments. In addition, the optimal surface nanotopography for selective adsorption of proteins that enable adhesion of osteoblastic cells and rapid bone apposition remains to be elucidated. Therefore, other roughening methods with more predictable outcomes are required.

PIIID overcomes the limitations of regular ion beam implantation and is suitable for processing samples with complex shapes (20). Following PIIID processing, the anti-corrosion, anti-abrasion and anti-fatigue properties of materials are significantly improved and therefore, it has gained attention worldwide and is now used in several biomedical applications (11–13). In the present study, PIIID was used to implant and deposit Zn ions within and onto the smooth surface of cp-Ti.

According to XPS analysis, with increasing Zn ion implantation and deposition time, carbon, Ti and Zn signals increased on the Zn-PIIID-Ti surfaces, whereas oxygen signals decreased. The significant Zn content on the surface reached its maximum with the 80 min deposition time. Following implantation and deposition, the surfaces contained ZnO and TiO_2_. SEM imaging revealed that following Zn-PIIID, dispersed lumps had formed on the originally smooth Ti surface and this may explain why certain Zn ions were implanted within the substrate matrix in the form of interstitial atoms, while other Zn ions deposited directly onto the Ti surface. Notably, the physical structure of the Zn-Ti-80 min sample surface was the most homogeneous among assessed surfaces, indicating that the size of granular masses became more uniform with increasing PIIID processing time. SEM, in turn revealed that Ti surfaces exhibited a rough ‘honeycomb’ structure with a diameter range of 60–100 nm upon PIIID processing. The ‘honeycomb’ structure continually deepened with increasing PIIID processing time and reached a depth of 200 nm with grade 9 smoothness and 0.4 *μ*m roughness in the Zn-Ti-80 min group. Thus, the present study demonstrated that the PIIID technique for Ti surface modification offers an easy and effective way to increase the roughness of the surfaces of Ti implants and the degree of roughness achieved is more significant, with a deeper nanostructure, than that generated using other methods (6,21,22).

MG-63 cells were seeded onto pure and Zn-modified Ti surfaces in order to assess the effects of Zn implantation and deposition on the biocompatibility of the Ti surfaces. SEM images revealed pseudopodia extending from osteoblasts residing on the Zn-modified Ti surfaces, indicating active cellular metabolism. Cells extended multiple long and slender pseudopodia that inserted between the granules of the Zn-modified Ti surfaces and formed anchoring structures for strong adhesion. The present study also revealed that the density of attached cells on the Zn-modified surfaces was significantly greater than that observed on the unmodified Ti surfaces and the secretion of ECM proteins followed this trend as well, with ECM proteins covering the granules of Zn-modified Ti surfaces. The current findings are in agreement with the electron microscopy images presented in previous studies (14,23,24) of murine bone marrow cells grown on hydroxyapatite-coated cp-Ti surfaces following 2 h incubation.

Based on the collective results of the present study, it is concluded that Ti substrates modified using Zn PIIID exhibit improved biocompatibility with the MG-63 cell line compared with cp-Ti and thus may be applicable in a wide variety of dental implants. Experiments to determine the *in vivo* performance of these Zn-modified Ti substrates are underway. Based on the present results demonstrating the biocompatibility of these substrates as well as previous findings that Zn-modified Ti surfaces discourage bacterial adhesion (14), it is expected that Zn-modification of Ti may enhance the success rate of dental implants.

## Figures and Tables

**Table I:** X-ray photoelectron spectroscopy elemental percentages for Zinc-plasma immersion ion implantation and deposition on titanium surfaces.

Element	cp-Ti	Zn-Ti-20 min	Zn-Ti-40 min	Zn-Ti-60 min	Zn-Ti-80 min
Ti	62.76±3.24	65.44±3.98	64.61±3.55	61.61±3.61	57.51±3.47
O	37.24±1.26	33.52±1.33	34.08±1.19	37.04±1.38	40.35±1.41
Zn	0.00	1.04±0.08^a^	1.32±0.06^a^,^b^	1.35±0.04^a^,^b^	2.14±0.06^a^,^b^,^c^

a P<0.01, compared with the cp-Ti group;

b P<0.05, compared with the Zn-Ti-20 min group;

c P<0.05, compared with the Zn-Ti-40 min and Zn-Ti-60 min groups. TI, titanium; Zn, zinc. The results are presented as the mean ± standard deviation.
